# The challenge of measuring spinopelvic parameters: inter-rater reliability before and after minimally invasive lumbar spondylodesis

**DOI:** 10.1186/s12891-022-05055-9

**Published:** 2022-01-31

**Authors:** Marc Hohenhaus, Florian Volz, Yorn Merz, Ralf Watzlawick, Christoph Scholz, Ulrich Hubbe, Jan-Helge Klingler

**Affiliations:** Department of Neurosurgery, Medical Center - University of Freiburg, Faculty of Medicine, University of Freiburg, Breisacher Str. 64, 79106 Freiburg, Germany

**Keywords:** Sagittal balance, Sagittal alignment, Spinopelvic parameters, Minimally invasive, Spine surgery, Lumbar spondylodesis, Inter-rater reliability, Intra-class correlation coefficient

## Abstract

**Background:**

The common manual measurement technique of spinal sagittal alignment on X-rays is susceptible to rater-dependent variability, which has not been adequately considered in previous publications. This study investigates the effect of those variations in the characterization of patients receiving lumbar spondylodesis.

**Methods:**

General alignment parameters on pre- and postoperative X-rays were evaluated by four raters in 43 prospectively sampled patients undergoing monolevel spondylodesis. The Intra-class Correlation Coefficient (ICC) for each rater pair and all raters together was calculated for inter-rater reliability. For the operation-induced change of the sagittal alignment in every patient the Wilcoxon test was applied to compare for each rater separately.

**Results:**

The ICCs were “good” (>0.75) to “excellent” (>0.9) for all raters together and for 45 of the 48 single rater pairs (93.75%). All revealed a significant increase of the addressed segmental lordosis and disc height and no significant change for spinopelvic parameters and sagittal vertical axis from pre- to postoperative. The lumbar lordosis showed a significant increase through the operation of +2.5° (p = 0.014) and +3.7° (p = 0.015) in two raters and no difference for the other ones (+2.1°, p = 0.171; -2.2°, p = 0.522).

**Conclusions:**

The pre- to postoperative change of lumbar lordosis revealed different significance levels for different raters, although the ICCs were formally good. Accordingly, the evaluation by only one rater would lead to different conclusions. Due to this susceptibility of alignment measurements to rater-dependent variability, the exact evaluation process should be described in every publication and the consistency of significant results be validated through multiple raters.

**Trials registration:**

The trial was approved by the local ethics committee and listed at the national clinical trials register (DRKS00004514, date of registration: 08/11/2012).

**Supplementary Information:**

The online version contains supplementary material available at 10.1186/s12891-022-05055-9.

## Background

The evaluation of sagittal spinal alignment parameters in lumbar degenerative spine surgery is of extensive interest. Pathologic alterations are on the one hand partially responsible for the development of degenerative diseases and on the other hand relevant for treatment planning to reach an optimal outcome of affected patients [1–3]. Diverse surgical strategies imply variable modification options of the spinal alignment, so that a preoperative evaluation is clearly recommended [2, 4]. The extent taking these parameters into account for the individual treatment strategy is discussed controversial.

The radiological assessment is usually still done manually on plane X-ray images, which is prone to rater-dependent variation [5]. The dimension depends on the parameter and is reported up to an average of 10° for spinopelvic and lumbar lordosis angles [6–8]. For thoracic and cervical parameters, variations of more than 10° are described and dependent on the body position [9]. Causative seem to be the subjective measurement technique itself as well as the image quality, the individual position and configuration of the patient [5]. There have been several imaging standardization attempts, e.g. by the use of whole spinal imaging systems, like the EOS^®^ system, with reported increases of image quality and reduced radiation exposure [10, 11]. Some studies reported advantages for software-based parameter evaluations, whereas a fully automated assessment still does not exist [6, 12, 13]. There is no “gold standard” for detecting the real value of the sagittal alignment and no clear recommendation for standardized evaluation of spinal alignment yet.

An impact of such an inter-rater variation on study outcomes can be expected. The discussion of this relevant bias within previous as well as recent publications is heterogeneous. Many authors refer no information on this issue or report data of single raters [14–26]. Only few publications include a more detailed statement of the number of raters and evaluation procedure but rarely with a clear statement of inter-rater reliabilities [6, 27–29]. The quality of each publication is narrowed through an absent detailed statement concerning the evaluation procedure and the associated variability. Nevertheless, the precise impact of these variations seems to be unclear, because the reported effect strength is often diminutive.

The purpose of this study is to evaluate the effect of inter-rater variations within the measurement of sagittal alignment parameters in pre- to postoperative characterization of patients with monolevel lumbar spondylodesis. Thus, the relevance of the Intra-class Correlation Coefficient (ICC) should be clarified.

## Methods

The aim of the study was to compare pre- and postoperative lumbar sagittal alignment parameters measured by different raters. We assumed that the variability of subjective measurements has a relevant impact on the significance levels of evaluated differences.

### Study population

Patient data were sampled within a prospective, single-center, single-arm cohort study [30]. The trial was approved by the local ethics committee and listed at the national clinical trials register (DRKS00004514, date of registration: 08/11/2012). The study was carried out in accordance with relevant guidelines and regulations. In total 50 patients were included in this prospective trial after giving informed consent to participate. Seven patients were excluded for our radiographic evaluation because of the treatment of two lumbar levels, resulting in 43 patients receiving a monolevel, minimally invasive transforaminal lumbar interbody fusion (TLIF) due to a degenerative disease.

### Radiographic Evaluation

All patients received directly preoperative and one year postoperative plane X-ray images of the whole spine to evaluate sagittal alignment. X-rays were performed in standardized patient comfortable standing position.

**Fig. 1:**
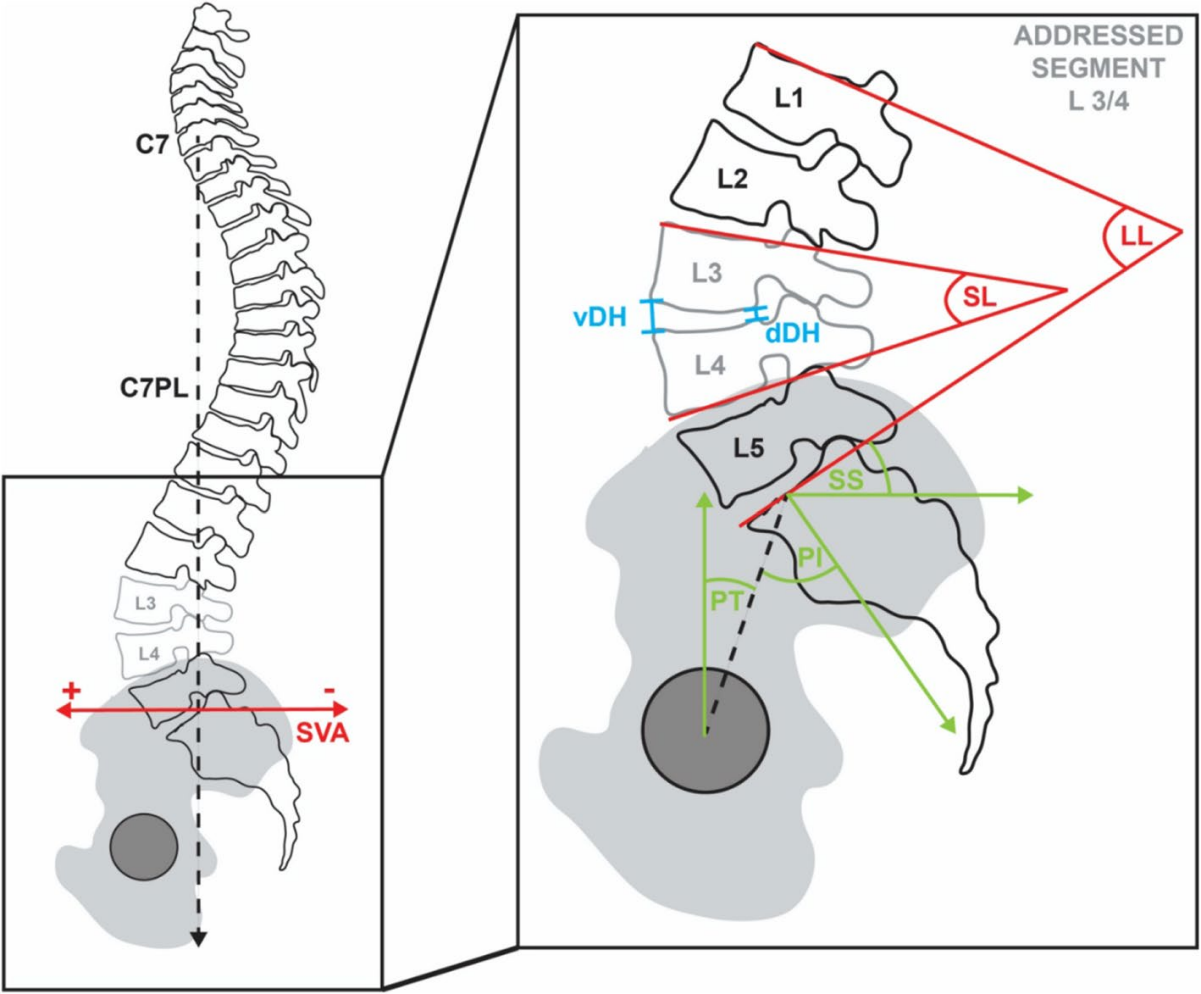
All evaluated lumbar sagittal alignment parameters: vDH = ventral disc height, dDH = dorsal disc height, SL = segmental lordosis, LL = lumbar lordosis, PI = pelvic incidence, PT = pelvic tilt, SS = sacral slope, C7PL = C7 plumb line, SVA = sagittal vertical axis

For evaluation of the sagittal lumbar alignment, the following parameters were measured: segmental lordosis (SL) as angle between superior endplate of the upper vertebral body and inferior endplate of the lower vertebral body of the addressed segment; ventral (vDH) and dorsal (dDH) disc height as distances of the ventral and dorsal edge of the treated vertebral disk; lumbar lordosis (LL) as angle between superior plate of L1 and S1; pelvic incidence (PI) as angle between the line of the center of the femoral heads to the center of the S1 endplate and the line orthogonal to the S1 endplate; pelvic tilt (PT) as angle between the line of the center of the femoral heads to the center of the S1 endplate and the reference vertical line; sacral slope (SS) as angle between S1 endplate and the reference horizontal line [7]. For global spinal balance the sagittal vertical axis (SVA) was measured as distance from the posterior superior corner of the S1 endplate to a vertical plumb line dropped from the center of C7 [2]. All parameters are depicted in Fig. 1.

### Outcome measurements

**Fig. 2:**
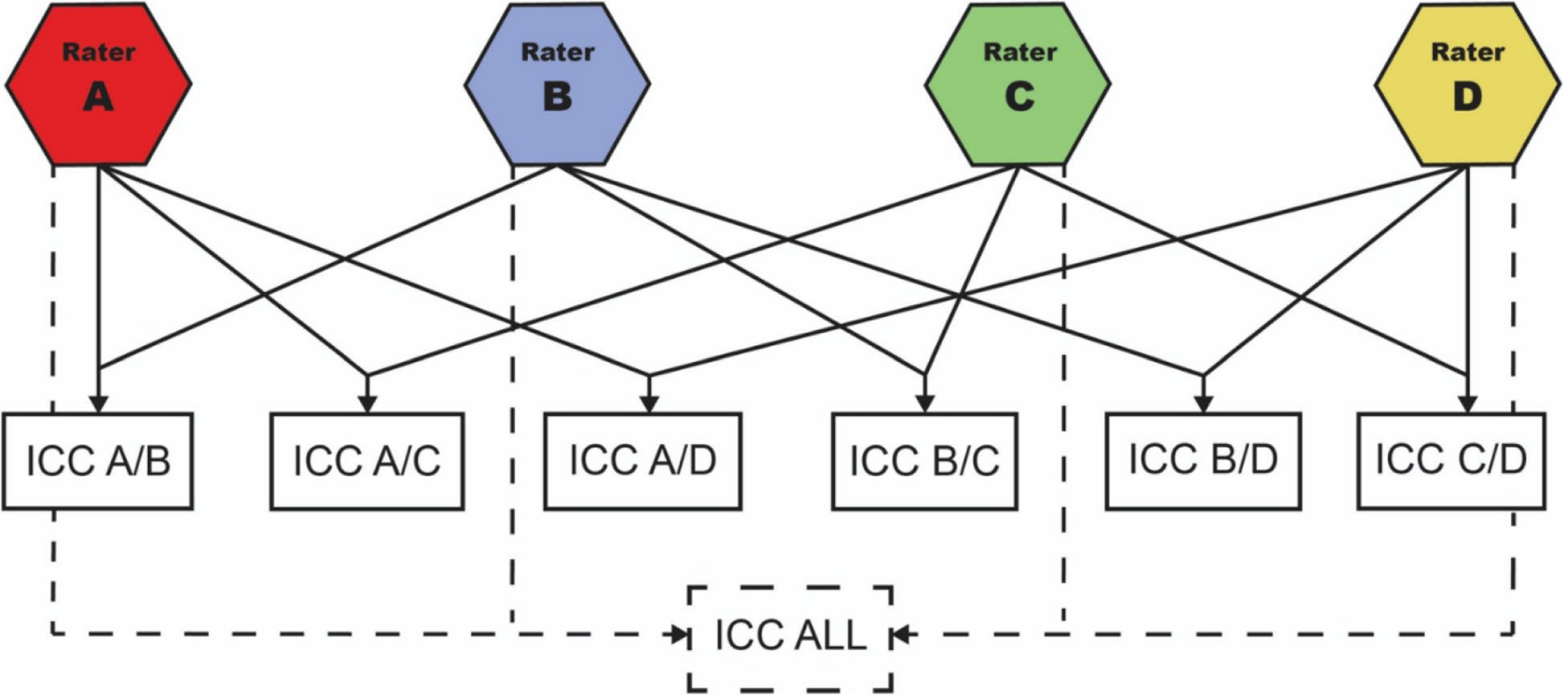
Overview of the different ICC calculations that were done for each sagittal alignment parameter

Four raters evaluated each alignment parameter: one spine surgeon with more than 30 years of experience (rater A), one senior physician with more than ten years of experience (rater B), one resident (rater C) and one postgraduate student who was instructed in the measurement (rater D). The measurements were done manually on digitized X-rays within IMPAX EE R20 (Agfa HealthCare©) and every rater was blinded to the results of the other ones. For the determination of the inter-rater reliability the ICC was computed for all possible rater pairs (“A/B”, “A/C”, “A/D”, “B/C”, “B/D”, “C/D”) and for all four raters together (“ALL”), resulting in seven ICC values for each spinal parameter (see Fig. 2).

As second step, we divided our measurement results associated to the treatment of all patients into pre- and postoperative values, respectively. The differences induced by the operation were compared for every parameter in each rater separately.

### Statistics

Data processing and statistical analysis were performed using IBM SPSS Statistics 25 and the R Project for Statistical Computing. Normal distribution for each variable was assessed by Shapiro-Wilk-Test. The inter-rater reliability for the radiographic outcome parameters was calculated using the ICC for absolute scales with multiple raters [31]. We calculated a two-way mixed-effects model, with single measures and absolute data agreement [32]. All ICC values were qualified according to Koo et al. [32] with an additional color-coding in Table 2: <0.50 = poor (red), 0.50 – 0.75 = moderate (yellow), 0.75 – 0.90 = good (green), >0.90 = excellent (blue).

For comparison of the single rater measurements pre- and postoperatively additionally to the ICC calculation, we added a one-way ANOVA calculation. Homogeneity of variances was asserted using Levene’s Test and revealed consistently equal variances (p > 0.05). Post-hoc-analysis was conducted using Tukey’s test.

We compared pre- to postoperative changes of each spinal alignment value within each rater and the mean of all raters together using the Wilcoxon test for paired samples. *P* values <0.05 were considered to be statistical significant.

## Results

### Baseline characteristics

Median age at the time of surgery of all 43 included patients was 57 years (interquartile range - IQR 48 - 69), 18 (41.9%) were male and 25 (58.1%) female. A spondylolisthesis was present in 42 patients with Meyerding grade I in 51.2% (n = 22) or grade II in 46.5% (n = 20). One patient showed no spondylolisthesis (2.3%). Primarily addressed level was L5/S1 (n = 22, 51.2%), followed by L4/5 (n = 17, 39.5%) and L3/4 (n = 4, 9.3%). Median Body-Mass-Index was 26.3 kg/m^2^ (IQR 23.1 - 28.7). All baseline characteristics are summed up in Table 1.

**Table 1:** Baseline characteristics of all 43 patients, ^a^Median (IQR)

Age	years	57 (48 - 69)^a^
**Gender**	female	25 (58.1%)
	male	18 (41.9%)
**Fused level**	L3/L4	4 (9.3%)
	L4/L5	17 (39.5%)
	L5/S1	22 (51.2%)
**Spondylolisthesis**	Meyerding grade I	22 (51.2%)
	Meyerding grade II	20 (46.5%)
**Body-Mass-Index**	kg/m^2^	26.3 (23.1 - 28.7)^a^

### Radiographic characteristics

Patients received pre- and postoperative sagittal standing X-rays, resulting in 86 measurements for each parameter. The median time between surgery and follow-up X-ray was 366 days (IQR 365 - 379). In two patients the postoperative X-rays were insufficient for measurement of the spinopelvic parameters and additional three patients had only lumbar standing X-rays after surgery. Therefore the postoperative evaluation of PI, PT and SS was possible in only 41/43 and SVA in 38/43 patients.

### Inter-rater reliability

All ICC values are shown in Table 2. The inter-rater reliability was “excellent” (>0.9) for all measurements taking all raters together, except for the dDH with an almost “good” result (0.833, CI 0.713 – 0.899). The SVA showed the strongest agreement (0.995, CI 0.991 – 0.997), followed by PT (0.992, CI 0.989 – 0.995) and PI (0.977, CI 0.966 – 0.984).

For the single rater comparisons, the majority of ICCs were “excellent” too (37/48, 77.1%). Nine ICCs showed a “good” result (18.8%), two a “moderate” (4.2%) and only one a “poor” correlation (2.1%). In all single inter-rater comparisons, the dDH was the worst parameter, whereas the SVA showed the best correlations.

Within the ANOVA comparison of the measured values of all four raters pre- and postoperatively, we found a statistically significant difference only for the dDH pre- and postoperatively (*p* < 0.001 and *p* = 0.003). The other alignment parameters showed no significant differences. All values are shown in Supplement 1. The post-hoc-analysis showed an isolated significant difference between rater A and B preoperatively (difference -1.488° (CI -0.456° - -2.521°), p = 0.001) as well as between the following rater pairs postoperatively: A/B (-3.835°, CI -5.176° - -2.494°, *p* < 0.001), A/C (-3.709°, CI -5.050° - -2.369°, *p* < 0.001), A/D (-2.428°, CI -3.769° - -1.087°, *p* < 0.001) and B/D (1.407°, CI 0.066° - 2.748°, p = 0.036). This matches to the lowest ICC values for the dDH.

**Table 2:**
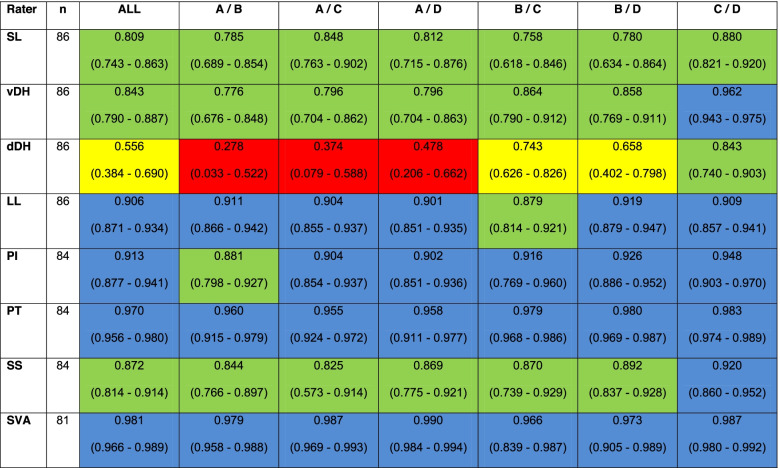
ICC calculations (95% CI) for all raters together and each rater pair. SL = segmental lordosis, vDH = ventral disc height, dDH = dorsal disc height, LL = lumbar lordosis, PI = pelvic incidence, PT = pelvic tilt, SS = sacral slope, SVA = sagittal vertical axis

Color-coding: ICC > 0.9 "excellent" in blue, ICC 0.75 - 0.89 "good" in green, ICC 0.5 - 0.74 "moderate" in yellow, ICC < 0.5 "poor" in red, according to Koo et al. [32]

### Comparison of pre- and postoperative parameters

The differences induced by the operation were compared for each rater separately (Table 3). All raters showed consistently significant higher SL angles as well as vDH and dDH. For spinopelvic angles (PI, PT, SS) as well as for the SVA no significant changes were detected. Interestingly, we found different statistically relevant values for the LL, resulting in a significant increased postoperative angle in two raters (A: +2.5°, *p* = 0.014; C: +3.7°, *p* = 0.015) and no significant difference within the other two raters (B: +2.1°, *p* = 0.171; D: -2.2°, *p* = 0.522).

**Table 3:** Pre- to postoperative differences of the sagittal alignment parameters for each rater separately. ^a^Median (IQR), SL = segmental lordosis, vDH = ventral disc height, dDH = dorsal disc height, LL = lumbar lordosis, PI = pelvic incidence, PT = pelvic tilt, SS = sacral slope, SVA = sagittal vertical axis. Significant values (*p* < 0.05) are marked in bold type

	preoperative^a^	postoperative^a^	Δ	p	preoperative^a^	postoperative^a^	Δ	p
Rater	Rater A				Rater B			
**SL**	19.0° (13.0 - 25.0)	24.0° (20.0 - 27.0)	+5.0°	**<0.001**	20.2° (15.5 - 24.3)	25.3° (18.3 - 30.9)	+5.1°	**0.001**
**vDH**	8.0mm (4.0 - 10.0)	13.0mm (11.0 - 14.0)	+5.0mm	**<0.001**	6.5mm (4.9 - 8.3)	13.5mm (11.9 - 15.2)	+7.0mm	**<0.001**
**dDH**	3.0mm (2.0 - 4.0)	5.0mm (4.0 - 6.0)	+2.0mm	**<0.001**	4.5mm (3.4 - 5.8)	8.4mm (7.0 - 10.2)	+3.9mm	**<0.001**
**LL**	57.0° (50.0 - 64.0)	59.5° (52.0 - 68.0)	+2.5°	**0.014**	57.0° (50.2 - 67.7)	59.1° (51.9 - 68.1)	+2.1°	0.171
**PI**	59.0° (50.0 - 68.0)	59.5° (52.8 - 69.0)	+0.5°	0.649	60.6° (56.0 - 69.6)	61.6° (54.1 - 67.8)	+1.0°	0.061
**PT**	20.0° (11.0 - 25.0)	19.0° (13.0 - 23.5)	-1.0°	0.231	20.9° (13.9 - 27.4)	20.6° (14.2 - 25.4)	-0.3°	0.251
**SS**	43.0° (37.0 - 48.0)	44.0° (38.8 - 49.0)	-1.0°	0.194	42.1° (36.2 - 45.9)	42.0° (35.9 - 47.1)	-0.1°	0.630
**SVA**	22.5mm (12.3 - 50.5)	25.0mm (9,5 - 43.5)	+2.5mm	0.751	24.9mm (14.6 - 47.6)	26.1mm (17.1 - 41.4)	+1.2mm	0.856
Rater	Rater C				Rater D			
**SL**	17.1° (11.8 - 22.4)	21.6° (18.8 - 26.6)	+4.5°	**0.001**	17.2° (13.2 - 22.7)	21.1° (18.2 - 26.3)	+3.9°	**0.012**
**vDH**	5.8mm (3.6 - 7.7)	13.6mm (12.3 - 14.8)	+7.8mm	**<0.001**	6.3mm (4.4 - 7.5)	13.2mm (11.2 - 14.0)	+6.9mm	**<0.001**
**dDH**	3.7mm (2.3 - 4.7)	9.1mm (6.5 - 10.3)	+5.4mm	**<0.001**	3.6mm (2.2 - 4.2)	7.4mm (5.1 - 9.5)	+3.8mm	**<0.001**
**LL**	53.8° (47.4 - 64.8)	57.5° (48.7 - 67.9)	+3.8°	**0.015**	59.3° (50.2 - 66.0)	57.1° (50.9 - 66.7)	-2.2°	0.522
**PI**	56.7° (51.4 - 70.2)	60.7° (48.9 - 68.8)	+4.0°	0.534	59.0° (52.1 - 73.2)	60.6° (54.1 - 68.9)	+1.6°	0.067
**PT**	20.3° (13.4 - 28.7)	18.6° (14.1 - 25.9)	-1.7°	0.273	21.3° (13.0 - 27.6)	19.3° (13.9 - 24.9)	-2.0°	0.202
**SS**	39.8° (31.9 - 44.8)	39.4° (32.7 - 47.5)	-0.4°	0.624	42.2° (36.0 - 48.7)	39.7° (34.6 - 47.3)	-2.5°	0.591
**SVA**	21.3mm (6.6 - 47.7)	24.7mm (6.6 - 39.8)	+3.4mm	0.983	22.0mm (13.1 - 46.0)	23.0mm (6.3 - 39.0)	+1.0mm	0.577

Finally, we calculated the mean values out of all four raters for each alignment parameter and compared the pre- to postoperative changes again, resulting in a significant increase of the LL of +1.4° (p = 0.035), whereas the other parameters showed similar significant tendencies like in all single rater evaluations (see Table 4).

**Table 4:** Pre- to postoperative differences of the sagittal alignment parameters for the mean values of all four raters together. ^a^Median (IQR), SL = segmental lordosis, vDH = ventral disc height, dDH = dorsal disc height, LL = lumbar lordosis, PI = pelvic incidence, PT = pelvic tilt, SS = sacral slope, SVA = sagittal vertical axis. Significant values (*p* < 0.05) are marked in bold type

	preoperative^a^	postoperative^a^	Δ	p
Rater	ALL			
**SL**	18.1° (13.7 - 24.2)	22.8° (20.5 - 28.4)	+4.7°	**<0.001**
**vDH**	6,5mm (4.3 - 8.4)	13.0mm (11.8 - 14.1)	+6.5mm	**<0.001**
**dDH**	3.8mm (2.6 - 4.8)	7.5mm (6.3 - 8.6)	+3.7mm	**<0.001**
**LL**	56.9° (48.9 - 64.9)	58.3° (51.3 - 68.3)	+1.4°	**0.035**
**PI**	58.8 °(52.3 - 70.3)	61.8° (51.7 - 68.5)	+3.0°	0.081
**PT**	20.8° (12.6 - 27.4)	19.6° (14.0 - 24.4)	-1.2°	0.151
**SS**	41.9° (35.5 - 46.4)	40.8° (36.5 - 48.7)	-1.1°	0.936
**SVA**	20.8mm (12.6 - 48.6)	24.6mm (9.8 - 41.3)	+3.8mm	0.862

## Discussion

In our cohort, the inter-rater reliability represented by the ICC was predominantly “good” or “excellent” between all raters for the measurement of sagittal spinal alignment parameters. In spite of the generally good agreement of all raters, there were different significance levels for the change of the LL from pre- to postoperative in patients receiving a monolevel, minimally invasive TLIF, which in summary leads to uncertainty concerning the estimation of these results.

The major problem is that we do not know the true value, because we refer to subjective measurements, which additionally depends on the heterogeneous image quality. There is no “gold standard” for determining the true value. Many publications only offer one rater for sagittal alignment measurements [14–26]. This seems to be critical because our evaluation showed that the results are rater-dependent and this can change the significance level, so that different raters could come to different conclusions. Taken only the results of rater A or C into account, we might postulate that the LL gets significantly increased through the minimally invasive TLIF, which could be classified as preferable result after this surgery technique. On the other hand, taken the rater B and D into account, no significant change and therefore benefit for the LL through the operation would be postulated.

Some prior studies report inter-rater variations, with mostly “good” or “excellent” ICC values [6, 29]. However, those values, formally reflecting a distinguished inter-rater reliability, may lead to a false sense of security. We could show that even with “good” or “excellent” ICCs in some cases the pre- to postoperative comparisons of each single rater showed different significance levels. This must be taken into account for the interpretation of previous as well as for future studies in the topic of sagittal alignment.

The variability of measurements seems to be independent from the formal “rater expertise”. Our evaluation showed that both senior raters (A and B) came to different results, as well as the two less experienced raters (C and D). Within the clinical practice the parameters for precise surgery planning are predominantly utilized through the experienced surgeons, respectively. But the rater experience in pervious published studies remains often unclear. We could not find a distinct difference concerning the experience as influencing factor in our evaluation.

### But how to find the true value?

In our opinion, the data quality can be improved if several raters work on the same data set. It has to be postulated that the higher the number of raters, the better the reliability. To find out how many raters are adequate to reach an acceptable certainty for every parameter remains a statistical challenge. This seems not only to be important for the evaluation of sagittal alignment parameters, but might also be relevant for other manually medical measurements. To determine the minimum count of raters is a future challenge for the statistics and under investigation now. A specific statistical procedure should be developed also taking the magnitude of the effect of the addressed parameter into account. Another solution would be the development of a fully automated evaluation software. Unfortunately there are no such software solutions for sagittal balance parameters yet. There are many software-supported calculations, like the KEOPS®, mediCAD Spine® or Spineview® software, but the key points like S1 endplate or the femoral heads have to be marked manually, which leads back to the problem of rater-dependent variations.

If the ICC calculation of several raters shows “good” to “excellent” results, the measurements generally seem to be reproducible. But our evaluation shows that it is not adequate to rely on this information. As second step when calculating differences between two samples, the statistical evaluation should be done additionally for every rater separately. If the significance levels are reproducible too, the reliability on the accuracy of the results is strengthened. If the significance levels differ, the results have to be handled with caution. Within our cohort, all raters showed similar significant differences for SL, vDH and dDH from pre- to postoperative. Therefore, we can assume that there is probably a underlying effect of the operation. From the spine surgeon point of view this result is perspicuous because of the implanted intervertebral spacer that elevates the disk space and the SL because of the intraoperative dorsal compression. The spinopelvic parameters as well as the SVA showed no significant changes consistent through all raters. This seems to be plausible too, because of monolevel, minimally invasive TLIF procedures have been performed without the goal of a significant alteration of the whole spinal sagittal balance. Crucial seems to be the LL because of different significance levels through different raters. According to that, the statement of a change of the LL through the minimally invasive TLIF must be handled with caution.

To calculate the mean values of the measurements of all raters for the comparison pre- to postoperative might be another solution to increase reliability of the findings by manual measurements (Table 4). This could increase precision, the more raters have participated. For our evaluation we have to postulate, that for the mean of all four raters the minimally invasive TLIF significantly increased the LL about +1.4° (*p* = 0.035). The other parameters showed consistent significance levels for the mean of all raters like in every single rater separately (Table 4).

A major limitation when evaluating alignment parameters is the heterogeneity of the single X-ray examinations resulting in a different image quality. This depends on the examiner, the position and the individual anatomy of each patient. A comparison with standardized whole spine X-rays, like the EOS® system, would be interesting, but was not part of this evaluation. Additionally a comparison to software-supported measurement methods would be interesting too. Unfortunately, there is no full-automatic computed evaluation program. Furthermore our study is an exclusively radiographic evaluation without associated clinically effects of our measurements.

## Conclusions

There was a “good” to “excellent” inter-rater reliability for the most sagittal alignment parameters. All raters detected a consistently significant increase of SL, vDH and dDH and no significant change of the PI, PT, SS and SVA after the operation. Nevertheless the pre- to postoperative change of the LL revealed different significance levels for different raters, although the ICCs were formally sufficient. Accordingly, the evaluation by only one rater would lead to different conclusions: Rater A and C would postulate a significant increase in LL following minimally invasive TLIF, while rater B and D would not detect any significant change.

We conclude that due to the susceptibility of spinopelvic parameter measurements to rater-dependent variability, several actions are recommended to increase reliability when evaluating significant changes: The measurements should be performed by several raters and the agreement should be statistically determined by ICC calculation. Even if the reproducibility is formally excellent, a validation of the consistency of the results in each rater should be included. In case of inconsistent levels of significance, the results should be handled with caution. Further investigations in statistical procedures are needed for the evaluation of subjective measured sagittal alignment parameters.

## Supplementary Information

**Additional file 1.**

## Data Availability

The datasets used and/or analysed during the current study are available from the corresponding author on reasonable request.

## Abbreviations

C7PL: C7 plumb line
dDH: dorsal disc height
ICC: Intra-class Correlation Coefficient
IQR: interquartile range
LL: lumbar lordosis
PI: pelvic incidence
PT: pelvic tilt
SL: segmental lordosis
SS: sacral slope
SVA: sagittal vertical axis
TLIF: transforaminal lumbar interbody fusion
vDH: ventral disc height
