# Supplementary material for: The challenge of measuring spinopelvic parameters: inter-rater reliability before and after minimally invasive lumbar spondylodesis
Source: BMC Musculoskelet Disord. 2022 Jan 31;23:104. doi: 10.1186/s12891-022-05055-9 (PMC8802499; doi:10.1186/s12891-022-05055-9)
Supplement: Supplementary file 1 — Additional file 1. [file 12891_2022_5055_MOESM1_ESM.docx]

**Additional file 1**

|  | **preoperative** | | **postoperative** | |
| --- | --- | --- | --- | --- |
|  | **F** | **p** | **F** | **p** |
| **SL** | F(3,168) = 0.496 | 0.686 | F(3,168) = 1.404 | 0.243 |
| **vDH** | F(3,168) = 1.753 | 0.158 | F(3,168) = 1.130 | 0.338 |
| **dDH** | F(3,168) = 4.963 | **0.003** | F(3,168) = 23.719 | **<0.001** |
| **LL** | F(3,168) = 0.359 | 0.783 | F(3,165) = 0.067 | 0.977 |
| **PI** | F(3,168) = 0.670 | 0.572 | F(3,157) = 0.211 | 0.889 |
| **PT** | F(3,168) = 0.195 | 0.899 | F(3,157) = 0.100 | 0.960 |
| **SS** | F(3,168) = 0.853 | 0.467 | F(3,157) = 1.327 | 0.268 |
| **SVA** | F(3,167) = 0.119 | 0.949 | F(3,147) = 0.617 | 0.605 |

Significance levels for ANOVA testing for pre- and postoperative measurements comparing all four raters. SL = segmental lordosis, vDH = ventral disc height, dDH = dorsal disc height, LL = lumbar lordosis, PI = pelvic incidence, PT = pelvic tilt, SS = sacral slope, SVA = sagittal vertical axis. Significant values (p<0.05) are marked in **bold type.**
